# Weak Magnetic Fields Enhance the Efficacy of Radiation Therapy

**DOI:** 10.1016/j.adro.2021.100645

**Published:** 2021-01-16

**Authors:** Keisuke S. Iwamoto, Robert E. Sandstrom, Mark Bryan, Yue Liu, S. Robin Elgart, Ke Sheng, Michael L. Steinberg, William H. McBride, Daniel A. Low

**Affiliations:** a Department of Radiation Oncology, David Geffen School of Medicine at UCLA, Los Angeles, California; b Triplet State Technology LLC, Longview, Washington; c Mark Bryan & Company LLC, Arcadia, California

## Abstract

**Purpose:**

The clinical efficacy of radiation therapy is mechanistically linked to ionization-induced free radicals that cause cell and tissue injury through direct and indirect mechanisms. Free radical reaction dynamics are influenced by many factors and can be manipulated by static weak magnetic fields (WMF) that perturb singlet-triplet state interconversion. Our study exploits this phenomenon to directly increase ionizing radiation (IR) dose absorption in tumors by combining WMF with radiation therapy as a new and effective method to improve treatment.

**Methods and Materials:**

Coils were custom made to produce both homogeneous and gradient magnetic fields. The gradient coil enabled simultaneous in vitro assessment of free radical/reactive oxygen species reactivity across multiple field strengths from 6 to 66 G. First, increases in IR-induced free radical concentrations using oxidant-sensitive fluorescent dyes in a cell-free system were measured and verified. Next, human and murine cancer cell lines were evaluated in in vitro and in vivo models after exposure to clinically relevant doses of IR in combination with WMF.

**Results:**

Cellular responses to IR and WMF were field strength and cell line dependent. WMF was able to enhance IR effects on reactive oxygen species formation, DNA double-strand break formation, cell death, and tumor growth.

**Conclusions:**

We demonstrate that the external presence of a magnetic field enhances radiation-induced cancer cell injury and death in vitro and in vivo*.* The effect extends beyond the timeframe when free radicals are induced in the presence of radiation into the window when endogenous free radicals are produced and therefore extends the applicability of this novel adjunct to cancer therapy in the context of radiation treatment.

## Introduction

The efficacy of conformal and intensity modulated radiation therapy is directly related to dose distribution conformality such that the ratio of tumor to normal structure doses is maximized. Dose distribution conformality is limited by ionizing radiation (IR) dose deposition physics, which is additionally influenced by the type and energy of the radiation. The vast majority of treatments are delivered using megavoltage photon beams, whose dose deposition properties are well understood.^1^, ^2^, ^3^, ^4^ For the past 20 years, increasingly sophisticated methods for planning and delivering these beams, with increasingly conformal radiation dose distributions, have been developed.^5^ Although this trend looks likely to continue with the advent of completely digital linear accelerators,^6^ even with current and future developments, x-ray dosimetry is limited by physics. Further increasing the relative tumor to normal tissue cell kill will require going beyond the physical dose distribution.

IR causes DNA damage and ultimately cell kill through the intermediate production of free radicals that largely stem from homolytic fission. These free radicals either recombine with minimal biological consequence or interact with intracellular molecules to induce biologically relevant responses. Damage to DNA damage is a major cause of IR-induced cell death. In addition, post-IR exposure–induced signaling cascades are initiated that further generate free radicals to amplify and perpetuate radiation effects.^7^ It follows that the free radical recombination kinetics are critical to the outcome of IR responses. These interactions can be altered by the presence of weak magnetic fields (WMF), a fact that has been used to modify free radical generation rates in theoretical and experimental molecular chemistry systems.^8^, ^9^, ^10^, ^11^ In brief, free radical pairs can be uncorrelated and spin-correlated, with electron spins randomly and mutually oriented, respectively. When the spins are aligned, the total spin angular quantum number, S, is 1, and 3 projection states for the spin quantum number M_s_ are possible: M_s_ = −1, 0, 1. Typically, mixing occurs of the singlet (S = 0) and triplet (S = 1) states, but the application of external WMF modifies the outcome by coupling to the M_s_ = −1 and M_s_ = 1 projections. These triplet radical pairs do not recombine and are a source of long-lived free radicals that can amplify primary and secondary free radical−mediated biological responses.

Growing evidence suggests that IR-induced cell death and tissue damage, whether in a tumor or normal organ, initiates from the free radicals directly formed by the deposition of covalent bond-breaking energy of IR but progresses owing to free radicals generated endogenously by the injured cell itself, challenging the current paradigm that damage is created solely during the irradiation.^12^ Mitochondria have been conjectured to be a major source of radiation-induced endogenously generated free radicals.^13^ In fact, tracking of mitochondrially generated hydroxyl radicals and superoxide anions demonstrated their transient increase postirradiation.^14^ Our data also suggest endogenous production of free radicals after irradiation.

Metabolically formed free radicals act as second messengers in cell signaling cascades that elicit changes in gene expression patterns.^15^, ^16^, ^17^ Such changes could function to orchestrate the availability of metabolic substrates during key steps in cellular function in response to environmental cues.^18^^,^^19^ One of the main themes in signal transduction is phosphorylation and dephosphorylation reactions; importantly, protein kinases are activated by oxidation whereas protein phosphatases and zinc finger proteins are inactivated by oxidation; transcription factor binding is enhanced by reduction reactions.^15^^,^^17^^,^^20^, ^21^, ^22^ Furthermore, aligned with their proposed signaling role, redox-sensitive changes to proteins are generally reversible, facilitating constant readjustments necessary to maintain a dynamic nonequilibrium steady state process of metabolic activity to match changes in cellular function.

Not surprisingly, therefore, free radicals have been proposed to regulate gene expression after IR by activating ATM, MAPKs, NRF2, NF-kB, and AP-1.^23^, ^24^, ^25^ These transducer proteins are part of the DNA damage response mechanism that senses DNA double-strand breaks and initiates a cascade of reactions that can determine the fate of the irradiated cell.^26^, ^27^, ^28^, ^29^

Here we demonstrate that the application of weak external static magnetic fields can increase the effective radiation dose in regions where the magnetic field and the radiation field coexist. We show that WMF of specified strengths are required to enhance IR killing of cancer cells and tumors in vitro and in vivo*,* without noticeable added toxicity.

## Methods and Materials

We evaluated the application of WMF on 4 of the most important signatures of cellular radiation damage: free radical production, DNA damage, in vitro clonogenic survival, and in vivo tumor growth.

### Cells

The cell lines, Lewis lung carcinoma (LLC) and human glioblastoma U87MG-viii, were maintained in RPMI1640 or DMEM (Mediatech Inc, Herndon, VA), respectively, supplemented with 10% fetal bovine serum (Omega Scientific, Tarzana, CA). The LLC cell line was purchased from American Type Culture Collection (Rockville, MD). The U87MG-viii cell line was kindly provided by a colleague (P.S. Mischel, University of California, San Diego).

### Animals

C3H/Sed/Kam or C57BL/6 mice were bred and housed within our departmental Association for Assessment and Accreditation of Laboratory Animal Care-accredited gnotobiotic mouse facility. All animal studies were approved by the Institutional Animal Care and Use Committee).

### Irradiations

A cobalt gamma-irradiator (Best Theratronics, Ontario, Canada; 1 Gy/min) at an SSD of 50 cm, or a Gulmay Medical RS320 x-ray unit operated at 300 kV (1.7 Gy/min; Gulmay Medical Ltd., Camberly, Surrey, UK) at an focus to surface distance of 34.7 cm was used.

### Magnetic field generation

**Figure 1:**
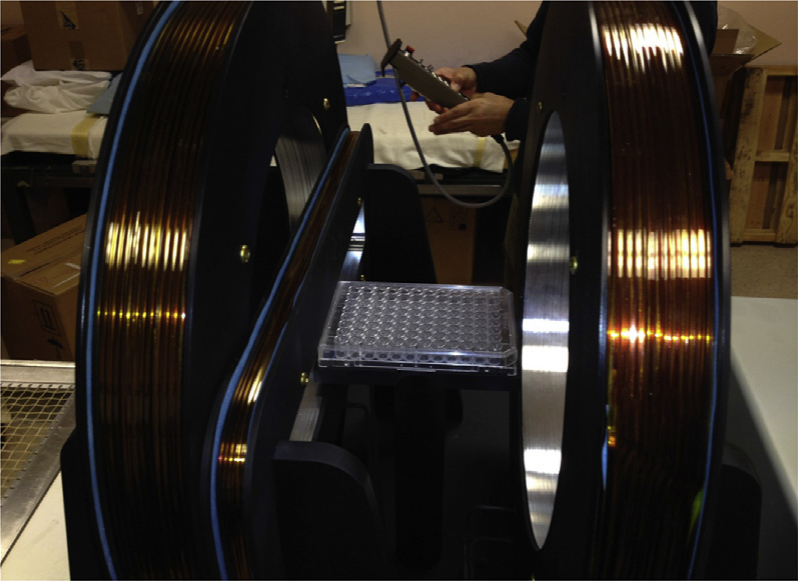
Gradient weak magnetic fields generating coils. This custom-built equipment produces a gradient of approximately 20 G across a 96-well plate (center) due to the single rectangular coil on the left. The 2 circular coils generate a homogeneous field that can be used to additively create a gradient from 1 to 100 G in increments of 20 G. *P* < .05 by Student *t* test.

A Helmholtz coil-based system was custom built (RadiaBeam Technologies, Santa Monica, CA) to provide a relatively homogeneous magnetic field (<1% heterogeneity) within a cylindrical volume with diameter 20 cm and length 20 cm (Fig 1). The magnetic field could be adjusted from 0 G to 40 G using a DC power supply (GW Instek, Montclair, CA). Some experiments required simultaneous irradiation with a range of magnetic fields, so a small gradient coil, based on a previous design,^30^ was integrated such that, when added to the homogeneous magnetic field, it provided an additive 0 to 26 G gradient across a standard 96-well plate during in vitro studies while the apparatus was placed in a nonmetallic 37°C incubator.

### Free radical measurement

The model for free radical production was reactive oxygen species (ROS) generation. Intracellular ROS were measured using 2’,7’-dichlorodihydro-fluorescein-diacetate (DCFH-DA) (Sigma-Aldrich, St Louis, MO), a nonfluorescent cell permeant that becomes trapped upon conversion into DCFH by intracellular esterases. The DCFH-DA becomes fluorescent on reaction with free radicals. For the ROS measurements cells were preincubated with 50 uM DCFH-DA. They were plated in 96-well plates and irradiated to 0 Gy, 2 Gy, 4 Gy, or 6 Gy (similar to current stereotactic body radiation therapy fraction doses) using ^60^Co gamma radiation while in a DC magnetic field of 0 G or 15 G. The magnitude of free radical production was measured using a 96-well plate fluorimeter (SpectraMax, Molecular Devices, San Jose, CA). Each experiment was repeated 3 times.

### DNA damage measurement

DNA damage was characterized in cells according to our previous studies that showed quantification of DNA damage via measurement of γ-H2AX using flow cytometry was equivalent to counting individual foci in a selected number of cells under a microscope.^31^^,^^32^ Briefly, the cells were placed in microfuge tubes and irradiated to 0 Gy or 1 Gy using ^60^Co gamma radiation or 300 KV x-rays with or without simultaneous exposure to magnetic field strengths ranging from 10 to 24 G. Thirty minutes after irradiation, when the greatest sensitivity to differences in DNA damage could be assessed, the cells were fixed, stained with FITC-labeled anti-γ-H2AX according to the manufacturer’s instructions (EMDMillipore, Burlington, MA), and assessed for DNA double-strand breaks using flow cytometry. Each experiment was repeated 3 times.

### Cell survival assessments

The effect of WMF on clonogenic survival was studied first using LLC cells. The cells were placed in 96-well plates and irradiated to 4 Gy and a range of magnetic fields from 6 G to 66 G. The cells were then plated and incubated for 2 weeks at 37°C, stained, and enumerated. Separately, U87MG-viii cells were irradiated to 4 Gy and at 0 G and 15 G. In addition to the standard irradiation sequence, where the cells were removed after irradiation, the cells were left in the magnetic field for 15 minutes after irradiation to determine whether the increased time in the magnetic field effected cell survival. For this experiment, the cells were placed at 37°C throughout the experiment. All experiments were repeated 3 times.

### In vivo assessments

Lastly, the effect of WMF on tumor growth was measured in 2 mouse models, C57BL/6 and C3H, representing syngeneic and allogeneic implantations, respectively. The eight 4- to 6-week-old female mice per group were subcutaneously engrafted with LLC cells (5 × 105) in the right leg flank and 4 days later exposed simultaneously to 0 Gy or 6 Gy and magnetic field strengths of 0 G, 12 G, or 38 G. Animals were anesthetized with a solution of ketamine (100 mg/kg) and xylazine (5 mg/kg) and one leg, implanted with the tumor cells, was isolated in the Co-60 field and locally irradiated. Exposure to WMF was whole-body. The WMF was maintained after the cessation of IR for a total WMF exposure time of 30 minutes. The growth of the tumors was measured using calipers daily between 5 and 15 days postimplantation in C57BL/6 mice and until 24 days in C3H mice. The time it took for the tumor to grow to 900 mm^3^ was used to define the tumor growth delay in the C57BL/6. For the C3H mice, the tumors were measured using calipers daily until they regressed to unmeasurable volumes or until the a priori determined temporal endpoint of 24 days, whichever occurred first.

## Results

**Figure 2:**
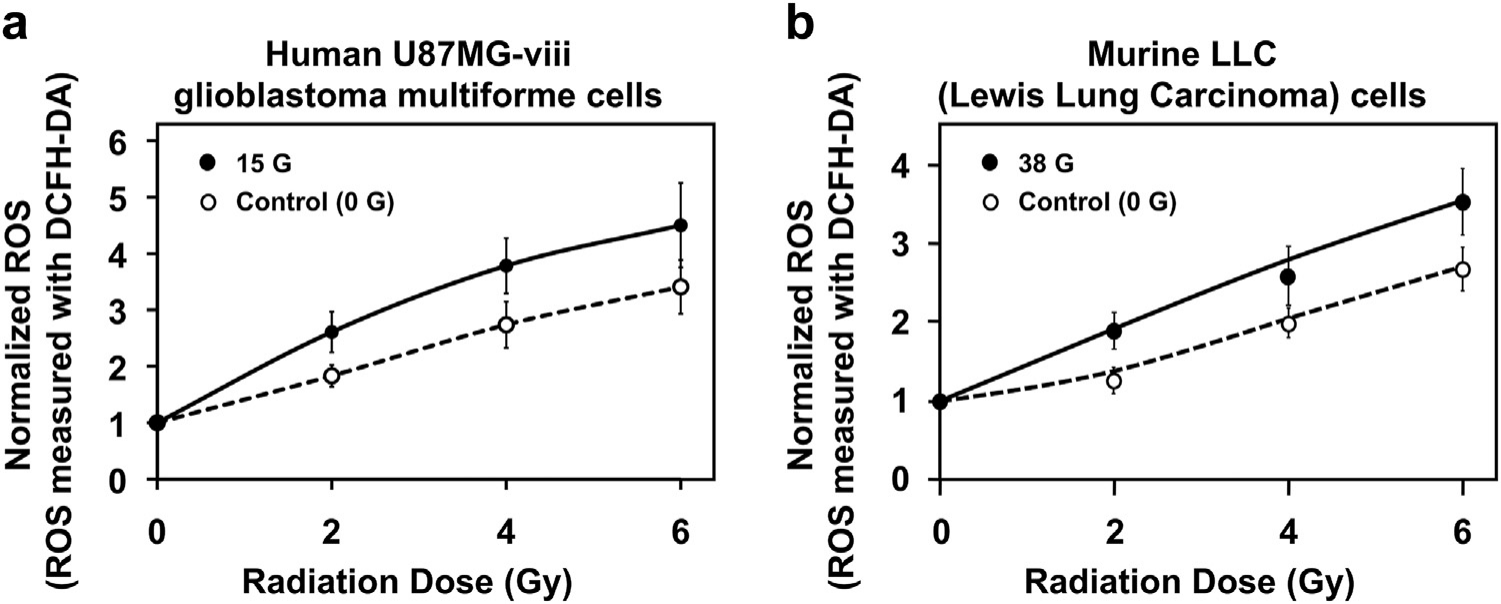
Free radical generation in cells. (a) U87MG-viii or (b) Lewis lung carcinoma cells were loaded with DCFH-DA and irradiated with or without simultaneous exposure to 15 or 38 G weak magnetic fields. *P* < .05 by Student *t* test. *Abbreviations*: DCFH-DA = 2’,7’-dichlorodihydro-fluorescein-diacetate; ROS = reactive oxygen species.

Application of WMF caused a systematic increase in the ROS production rates (*P* < .05), with an approximate 30% increase when coupled with 6 Gy in both the U87MGviii and LLC cells at 15 G and 38 G, respectively (Fig 2). The ROS concentrations were normalized to the unirradiated samples in the corresponding magnetic fields.

**Figure 3:**
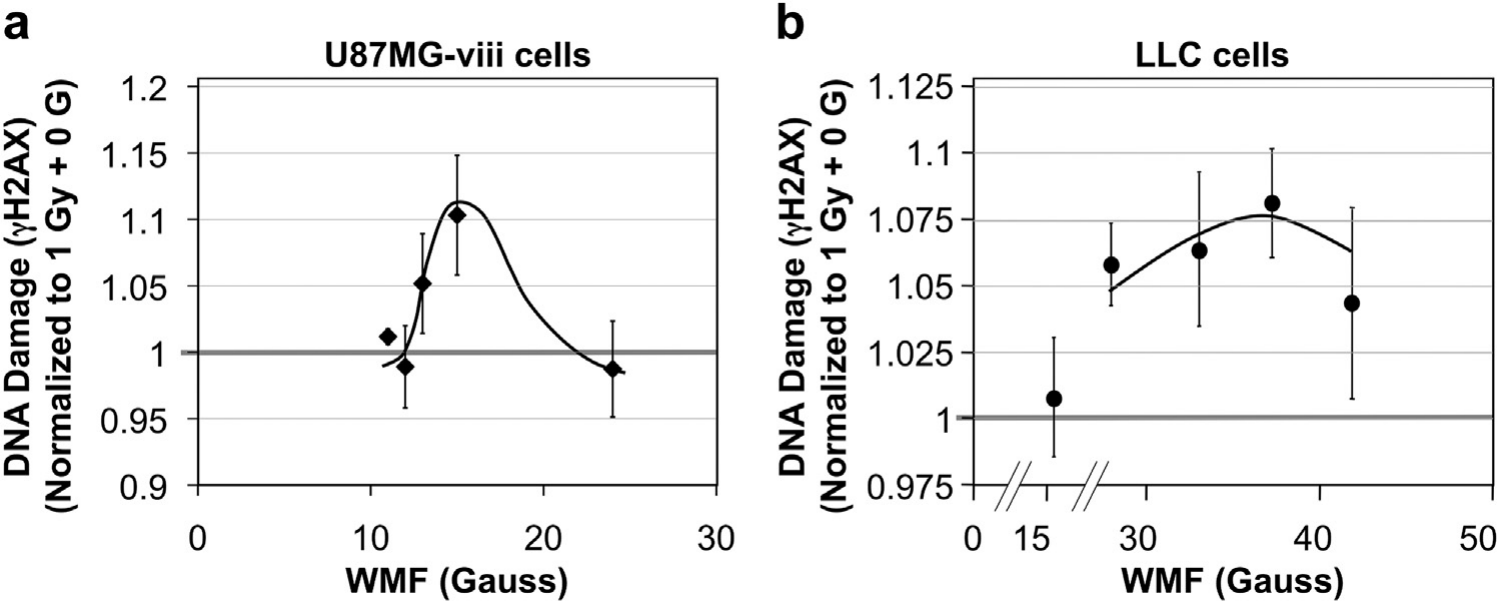
DNA damage in cells. (a) U87MG-viii or (b) LLC cells were exposed to 1 Gy with a range of WMF during and for 30 minutes postirradiation. Cells were incubated for a further 30 minutes and fluorescently labeled with anti-γ-H2AX for enumeration on a flow cytometer. *P* < .05 by Student *t* test. *Abbreviations*: LLC = Lewis lung carcinoma; WMF = weak magnetic fields.

The effect of the magnetic field on gamma radiation-induced DNA double-strand breaks, as estimated by γ-H2AX, in U87MGviii cells was most prominent near 15 G demonstrating an approximate 10% increase in signal compared with cells exposed to only the gamma radiation. In LLC cells, the largest enhancement of γ-H2AX observed was approximately 8% at 38 G (Fig 3)

**Figure 4:**
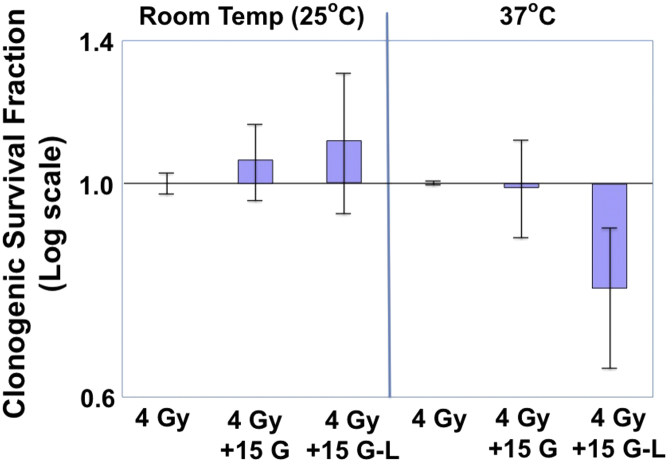
Clonogenic survival of U87MG-viii cells. Cells were irradiated with or without 15 G weak magnetic fields at 25°C or 37°C. Those irradiated in the weak magnetic fields were exposed to the 15 G only during the 4 Gy (4 Gy + 15 G) irradiation or during the 4 Gy irradiation plus 15 minutes; postirradiation (4 Gy + 15 G L). *P* < .05 by Student *t* test.

Using the optimal 15 G observed in the DNA double-strand break experiments, the results of irradiating U87MG-viii cells to 4 Gy and placing them in 0 G or 15 G for 0 or 30 additional minutes at either 25°C or 37°C are shown in Figure 4. At this field strength and for this cell line, the magnetic field did not enhance or decrease the radiation sensitivity unless the cells were kept in the magnetic field for an additional 30 minutes at 37°C, whereby the cell survival decreased by 20% ± 13%.

**Figure 5:**
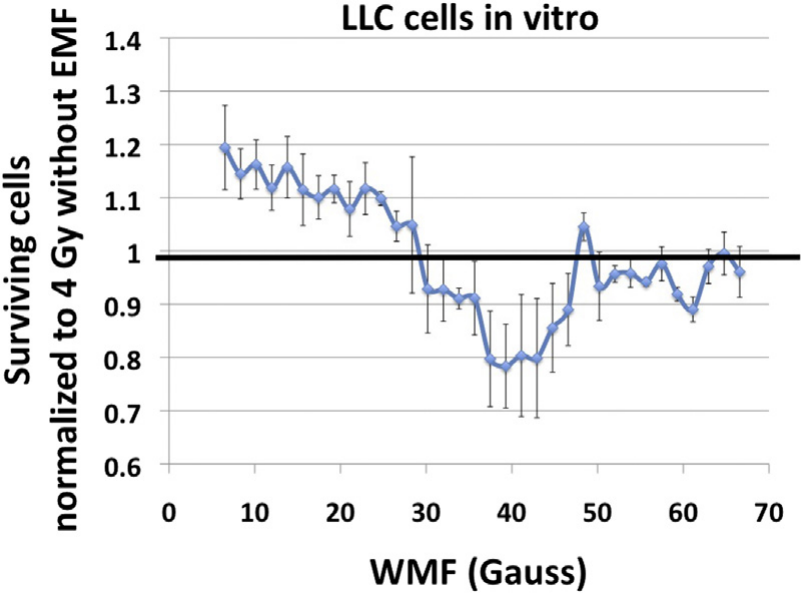
LLC survival using the gradient coils. Cells were grown in 96-well plates, placed in the apparatus shown in Fig 4, and irradiated with 4 Gy. *P* < .05 by analysis of variance. *Abbreviations*: LLC = Lewis lung carcinoma; WMF = weak magnetic fields.

In preparation for in vivo tumor studies in a normal immunocompetent environment, LLC cells, which will grow in wild-type C57BL/6, were assessed in vitro to verify that WMF affects them in a manner similar to that observed in radioresistant human U87MG-viii cells. LLC cells are also radioresistant and aggressively grow in syngeneic mice.^33^^,^^34^ Figure 5 shows the results of irradiating LLC cells to 4 Gy at a range of magnetic field strengths. A clear relationship is observed between cell survival and field strength; the largest reduction in survival (20%) is observed near 40 G, but an increase in survival of more than 10% is observed for fields less than 20 G.

**Figure 6:**
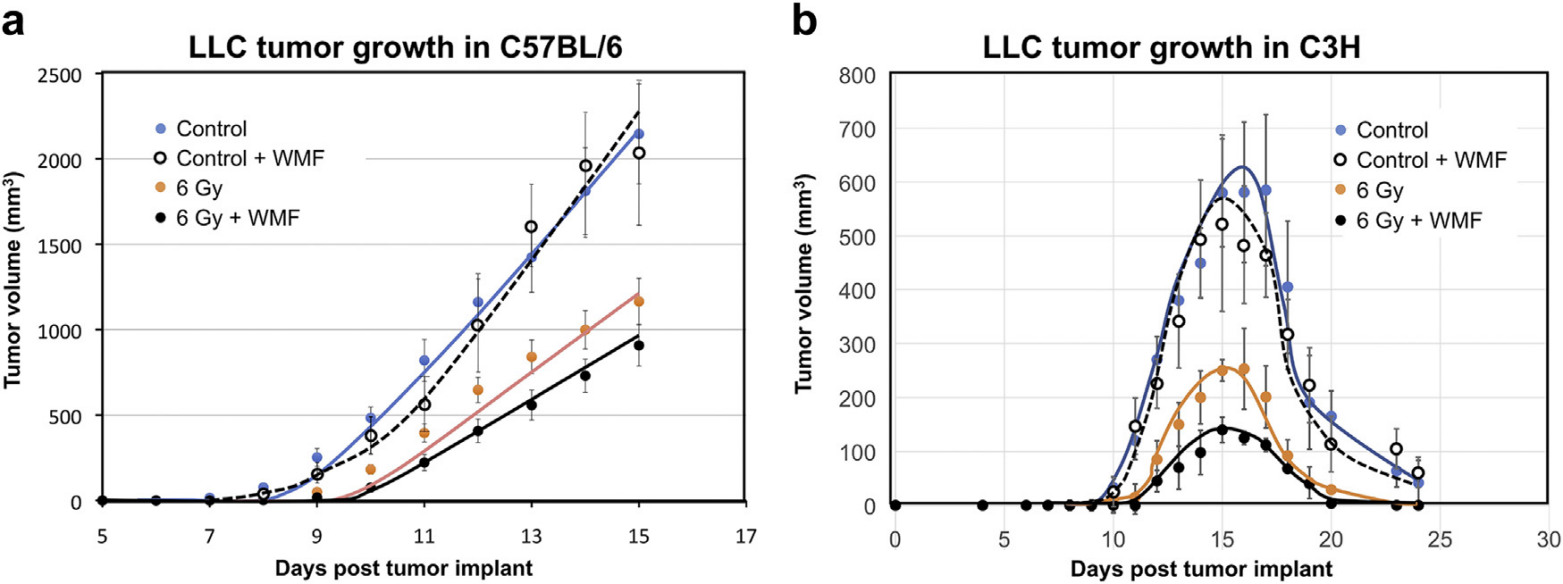
In vivo tumor growth and evidence for an immune component. LLC cells were grafted into the right leg of syngeneic (a) C57BL/6 or (b) weakly allogeneic C3H mice on day 4. All cohorts contained 8 animals. The legs were mock treated or irradiated with 6 Gy with or without exposure to 36 G weak magnetic fields (simultaneously plus 30 minutes postirradiation). Tumor growth was monitored by caliper measurements. *P* < .05 by analysis of variance. *Abbreviation*: LLC = Lewis lung carcinoma.

The in vivo tumor growth results are shown in Figure 6. The tumor growth delays (growth to 900 mm^3^) in C57BL/6 mice, where the LLC tumor is syngeneic, were 2 days and 3.75 days for irradiated and irradiated +38 G groups, respectively. In C3H mice, where the tumor was allogeneic, treated tumors grew with a 1- to 2-day delay compared with those in C57BL/6 mice, peaking in size on day 15 postimplantation, then regressing to undetectability by approximately day 24 regardless of treatment group. However, the maximum attained sizes were significantly different, with untreated controls reaching 600 mm^3^, 6 Gy-irradiated tumors reaching 250 mm^3^, and 6 Gy-irradiated plus 38 G tumors reaching 130 mm^3^. Mice exposed to WMF showed no demonstrably ill effects compared with control mice; furthermore, blood counts showed no difference in drop or recovery (data not shown).

## Discussion

Chemical reactions involving free radicals drive radiotherapeutic efficacy. Free radical recombination kinetics can be altered by the presence of very low-strength WMF,^35^ and we show that this phenomenon can be exploited to improve the therapeutic gain of radiation therapy. External application of a nontoxic WMF of specific strength increases free radical reactivity within the tumor, thereby increasing its radiosensitivity. Additionally, because metabolic free radicals stimulated by IR would also be affected, WMF alters cellular responses to IR-induced damage, DNA repair and survival, or DNA damage and death. Thus, we hypothesized that although tumors could be radiosensitized, normal tissues could be spared by WMF-mediated selective perturbation of free radicals involved in cell death and survival signaling, respectively, by means of customized field strengths at specific times post-IR. We present data showing WMF-dependent enhancement of IR-induced free radical production, DNA damage, in vitro cell kill and survival, and tumor shrinkage in a mouse model, without notable toxicity.

The main purpose of this study has been to introduce empirical evidence for the efficacy of static WMF as an adjunct to radiation therapy. Although other studies have reported on the physics of interactions between free radicals and WMF and the use of dynamic frequency-modulated electromagnetic fields in altering growth or response of cancer cells (using field strengths of at least 1 or 2 orders of magnitude higher than what we used), to our knowledge, we are the first to show that static WMF can significantly alter the free radical concentration after irradiation leading to enhanced effectiveness of radiation treatment of cancer. Therefore, we present a coherent picture based on an array of experiments that each demonstrates the ability of WMF to enhance the reactivity of IR-induced free radicals by limiting their recombination, which would modulate their cell-damaging abilities. First, we have shown using DCFH-DA in cells that WMF increases IR-induced free radical generations by as much as 40% at a clinically relevant dose of 2 Gy. This effective increase in free radicals translates to a relative dose enhancement factor of 2; that is, the level of DCFH-DA-measured free radicals after 2 Gy plus WMF is equivalent to 4 Gy without WMF. Ideally, such enhancement occurring solely in a tumor would double the therapeutic ratio.

The effects of WMF-enhanced free radical reactivity were evaluated by quantifying DNA double strand breaks, which are critical biological indicators of IR damage. There is a clear increase in γ-H2AX when the IR is accompanied by WMF. Moreover, the enhancement is dependent on the WMF field strength with optimal effects at around 15 G for the U87MGviii cells. Examination of a different cell line, LLC, indicated an optimal field strength distinct from that of U87MGviii cells and suggests that cell- or tissue-specific sensitivities to WMF could permit the simultaneous enhancement of radiation effects in tumors and mitigation in normal tissues. Part of this phenomenon could be because coupling of the triplet states depends on the chemical species associated with the free radical as well as the applied external WMF strength.^36^^,^^37^ These data suggest that first order perturbations to free radicals created by ionizing photons are not the sole effectors of the observed phenomenon. Effects on second order and third order free radicals formed as a cellular response to the initial IR-induced radicals implies a larger target for WMF manipulation of the radiotherapeutic effect on both the tumor and normal tissues.

The importance of free radicals induced by cellular responses to IR as a target for WMF manipulation is further supported by our experiments showing enhancement of cell death after prolonged exposure post-IR to WMF at a temperature of 37°C but not at 25°C, where metabolic rates and therefore production of endogenous free radicals are reduced. IR can generate free radicals through several biological processes, but the most important is of ROS from mitochondria, mainly superoxide, which can then be converted to hydrogen peroxide via superoxide dismutase.^38^, ^39^, ^40^, ^41^ Hydrogen peroxide and its potential byproduct hydroxyl radical can damage DNA or other cellular macromolecules or act as a signal transducer through oxidation of target molecules. Superoxide itself can also alter redox and mediate some cellular signaling. Perturbation of the temporal kinetics of these damaging/signaling radicals using WMF has the potential to alter subsequent cellular responses to damage control. We have preliminary data (not shown) where r^o^ U87MGviii cells, which lack mitochondria, lose their sensitivity to WMF-enhanced IR death, supporting the idea of WMF effects mediated by cellularly produced endogenous free radicals.

Finally, our in vivo tumor studies further corroborate the efficacy of combined radiation therapy and WMF. First, the decreased growth of LLC tumors in syngeneic C57BL/6 is evident when IR is combined with WMF compared with IR alone. In an allograft model of LLC in C3H mice, the significant reduction in maximum tumor size before regression, with little change in growth latency, suggests that WMF modulates immune response, enhancing antitumor activities. Clearly, free radicals, including ROS and reactive nitrogen species, are a critical component in the generation of immune sufficiency, particularly in inflammatory pathways. Studies have demonstrated that alterations of ROS- and reactive nitrogen species–generating capacity modulate activity of neutrophils, macrophages, and lymphocytes in response to both pathogens and tumor cells.^42^^,^^43^ Future studies will examine immunity markers, such as tumor-infiltrating cytolytic CD8 lymphocytes, that may be influenced by WMF to empower the immune response.

## Conclusions

Much of the clinical efficacy in radiation therapy results from chemical reactions involving free radicals that have short half-lives due to reactivity and recombination events. Free radical recombination kinetics can be altered by the presence of static low-strength WMF, and we demonstrate that this modulation can be exploited to increase the efficacy of radiation therapy. Our in vitro data indicate that WMF of any of the tested strengths from 6 to 66 G had no biological consequences alone but, when combined with IR, could enhance the generation of ROS, DNA double-strand breaks, and cell killing. We found that a restricted range of WMF strengths is optimal for combining with IR, depending on the biological system. Prolonging WMF exposure after IR for a total of 30 minutes enhanced the effectiveness of the combination and incubation at 37°C was essential. This result strongly implies that secondary free radicals generated through IR-induced biological cascades are a primary target for WMF enhancement of IR cytotoxicity.
